# 5G Numerologies Assessment for URLLC in Industrial Communications

**DOI:** 10.3390/s21072489

**Published:** 2021-04-03

**Authors:** David Segura, Emil J. Khatib, Jorge Munilla, Raquel Barco

**Affiliations:** Department of Communications Engineering, University of Malaga, 29071 Málaga, Spain; emil@uma.es (E.J.K.); munilla@ic.uma.es (J.M.); rbm@ic.uma.es (R.B.)

**Keywords:** 5G, numerology, URLLC, Industry 4.0, Industrial IoT

## Abstract

The fifth-generation (5G) network is presented as one of the main options for Industry 4.0 connectivity. Ultra-Reliable and Low Latency Communications (URLLC) is the 5G service category used by critical mechanisms, with a millisecond end-to-end delay and reduced probability of failure. 5G defines new numerologies, together with mini-slots for a faster scheduling. The main challenge of this is to select the appropriate numerology according to radio conditions. This fact is very important in industrial scenarios, where the fundamental problems are interference and multipath propagation, due to the presence of concrete walls and large metallic machinery and structures. Therefore, this paper is focused on analyzing the impact of the numerology selection on the delay experienced at radio link level for a remote-control service. The study, which has been carried out in a simulated cellular factory environment, has been performed for different packet sizes and channel conditions, focusing on outliers. Evaluation results show that not always a higher numerology, with a shorter slot duration, is appropriate for this type of service, particularly under Non-Line-of-Sight (NLOS) conditions.

## 1. Introduction

Traditionally, wired connections have been used in industrial networks. These networks connect the programmable logic controllers (PLC), i.e., the computers that control the machines, with each other and with the manufacturing execution system (MES). The MES usually contains process monitoring software, as well as alarm monitoring, and constitutes the interface between the PLCs and the enterprise resource planning (ERP), which allows a global coordination at executive level. The irruption of wireless technologies in industry enables new applications, as well as lower installation costs. At this time, the second, third and fourth generation (2G, 3G, 4G) networks coexist in commercial deployments and can cover some of the industry needs in a basic way, although not at the scale required for the most advanced applications. The fifth-generation (5G) network is a wide area network (WAN) that supports all communication profiles that occur in industrial scenarios.

In the Industry 4.0 paradigm, agility is a key objective in the design of factories. Some of the main technologies that enable such agility in factories are the following: rearrangeable modules in production lines, automated guided vehicles, autonomous robots, connected worker solutions and even drones. All these applications are critical and have a common requirement: a low latency. There are other Industry 4.0 applications with different sets of requirements, such as a high bandwidth, low power consumption or very high reliability, but this paper focuses on the problem of latency.

In recent years, there has been a huge involvement of the 3rd Generation Partnership Project (3GPP) members to define the fifth-generation access technology of mobile networks, better known as 5G New Radio (NR) [1]. The main objective is to provide flexibility to be able to work with a wide variety of bands and different use cases. 5G defines three types of services according to their requirements:

- Enhanced Mobile BroadBand (eMBB): high speed connections (up to 20 Gbps) and high traffic density.
- Massive Machine-Type Communications (mMTC): presence of many machine-type devices that through sporadic connections, exchange short messages over the network. It focuses primarily on the Internet of Things (IoT).
- Ultra-Reliable and Low Latency Communications (URLLC): critical communications, short and with very restrictive needs in latency and reliability. In particular, in 5G it is expected to reach a maximum transmission time of 1 ms at the user plane with a packet loss rate of $10^{-5}$ for a packet size of 32 bytes [2].

There are several approaches followed to achieve such requirements for URLLC. One technique is the reduction of the time-slot duration by means of a higher numerology [3]. These new numerologies have been defined in 5G, which are determined by a SubCarrier Spacing (SCS) and a cyclic prefix. Another solution consists of eliminating steps in the connection protocols to reduce the access time, known as Grant-free transmission [4]. In Pedersen et al. [5], some changes in the radio resource scheduler are proposed to allow multiplexing of eMBB and URLLC services, allowing a latency below 1 millisecond in the case of URLLC. In Rao and Vrzic [6], the authors study packet duplication over independent radio links as a means of achieving high reliability and low latency. In order to provide these independent links, several carriers are used that may come from the same or different base stations, where the user will be connected simultaneously. This is the concept of multi-connectivity, resulting from extending 4G dual-connectivity functionality to more than two base stations. In Khatib et al. [7], the authors study multi-connectivity technique for URLLC and the cost in throughput for other services, as eMBB services. In Patriciello et al. [8], the impact of the end-to-end delay is studied based on the choice of a numerology under Line-of-Sight (LOS) conditions in an urban macro scenario. However, the impact of the channel condition on the delay is not evaluated.

The aim of this work is to analyze the impact of the 5G numerology selection on the delay experienced in the radio link, more specifically, at Packet Data Convergence Protocol (PDCP) layer for a remote-control service, which needs a low latency target. In contrast to the studies described above, which are centered only under LOS conditions, an evaluation of different numerologies under LOS and NLOS (Non-Line-of-Sight) conditions is included, the last case being very frequent in industrial scenarios, where the interference and multipath propagation increases. The hypothesis in this paper is that although in LOS conditions a higher numerology implies a lower delay, this may not be fulfilled under NLOS conditions given its lower robustness. This will be a very important consideration in the design of 5G-based communications systems for URLLC in industrial scenarios.

The remainder of this paper is organized as follows. A brief description of new numerologies is presented in Section 2. The simulator alongside the scenario and the metric to evaluate the numerologies is described in Section 3. Results are shown in Section 4. Finally, conclusions are drawn in Section 5.

## 2. 5G New Radio Access Technology

New Radio (NR) access technology is based on a flexible orthogonal frequency division multiplexing (OFDM) system, which allows operating in a wide range of bands, addressing different use cases and operating under multiple spectrum access [3]. Regarding the waveform, OFDM with cyclic prefix is used as the downlink waveform for NR. In contrast to Long-Term Evolution (LTE), OFDM can also be used in the NR uplink and Direct Fourier Transform spread OFDM (DFT-s-OFDM), the last one with the aim of minimizing the Peak-to-Average-Power-Ratio (PAPR) [9]. NR Release-15 allows frequencies up to 52.60 GHz, defining two frequency ranges (FR): FR1 (410 MHz–7.125 GHz) and FR2 (24.25 GHz–52.60 GHz). Higher frequencies are considered for Release 16, still undefined. The maximum available bandwidth per component carrier is limited to 400 MHz and the maximum number of component carriers is 16.

### 2.1. Numerology Concept and Frame Structure

The NR frame structure can adopt different numerologies. A numerology is defined by a SubCarrier Spacing (SCS) and a cyclic prefix (normal or extended) [9]. Numerology (μ) can take values from 0 to 4, defining the SCS as $15\cdot 2^{\mu }$ kHz and the slot duration as $1/2^{\mu }$ ms, where high values of SCS are used at high frequencies. The maximum SCS value that can be reached is 240 kHz for μ = 4. However, not all numerologies are suitable for a frequency range. In the case of synchronization (PSS, SSS, PBCH), μ = {0, 1} is used in FR1 and μ = {3, 4} in FR2. On the other hand, in the case of data channels (PDSCH, PUSCH, among others), only μ = {0, 1, 2} is supported in FR1 and μ = {2, 3} in FR2 [10]. The number of subcarriers in NR is 12 for all numerologies. In order to maintain compatibility with LTE, frame duration is fixed at 10 ms and subframe duration at 1 ms. The number of slots per subframe is defined as $2^{\mu }$, depending on the selected configuration. Therefore, as numerology increases, there are more slots available but with shorter duration. One slot is composed by 14 OFDM symbols, so the OFDM symbol duration is 1/($14\cdot 2^{\mu }$) ms. Table 1 shows a summary of the characteristics for each numerology. An important remark is that μ = 0 corresponds to the legacy LTE configuration.

### 2.2. Mini-Slots

The scheduler usually performs transmissions at slot level. NR Release-15 defines the possibility of transmitting only a portion of a slot, with minimum value of two and one OFDM symbol in downlink and uplink, respectively. This is known as a mini-slot. These very short transmissions are used in situations that require very low latency, such as URLLC services.

### 2.3. Bandwidth Part

**Figure 1:**
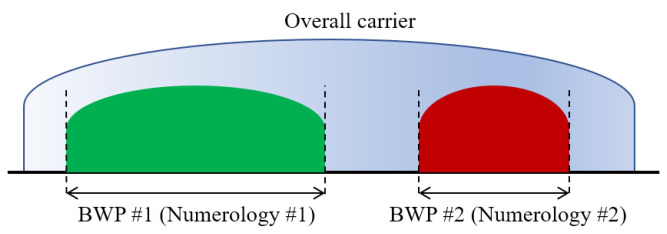
Frequency division multiplexing of numerologies.

Another feature included for NR is the Bandwidth Part (BWP) concept. BWP enable more flexibility in how resources are assigned in a given carrier. With BWP, a carrier can be subdivided and used for different purposes. Each BWP has its own parameters including bandwidth and numerology. Bandwidth is configured for each user equipment (UE) depending on its capabilities to support a maximum supported bandwidth and therefore, several UEs that have different capabilities can be served on a single broadband NR bearer. Moreover, multiple BWPs with different numerologies can be multiplexed within an NR bearer to support different types of services, as Figure 1 shows. Finally, an adaptation of the BWP based on changes between BWP with the same bandwidth and/or numerologies is also supported, with a single BWP being active at one time.

## 3. Methodology

In this section, the simulator used to carry out the evaluation performance of the different 5G numerologies is presented. Furthermore, the scenario simulated is presented as well as the method and metric used to evaluate the delay.

### 3.1. Simulator

To simulate a 5G mobile network, ns-3 has been used, a free and open-source network software simulator, very popular in research [11]. In particular, to recreate 5G cellular communication, there are two extended modules, which are based on an evolution of the ns-3/LENA module for LTE networks [12]:

- Millimeter-wave (mmWave) module [13]: implements the full 3GPP protocol, where the physical and media access control (MAC) layers are own implementations to support a new mmWave-based channel along with beamforming techniques and antenna models. The MAC layer supports time division duplex (TDD), and the scheduler is based on time division multiple access (TDMA). The rest of the upper layers are based on the functionalities of the LTE module, but with extensions such as dual connectivity and low latency in the radio link control (RLC) layer. Finally, it should be noted that the frame structure is not-based at slot level.
- 5G-LENA module [14]: this module is based on the mmWave module, focusing on the new 3GPP NR specifications and includes numerology support, frequency division multiplexing of numerology and an OFDMA-based scheduler. Unlike the mmWave module, the frame structure in the time domain and the scheduler have slot granularity, adapted as indicated by the standard for each numerology.

For this study, the 5G-LENA module has been selected to carry out the simulations, due to the slot granularity in the frame structure, as explained above.

### 3.2. Simulation Scenario

**Figure 2:**
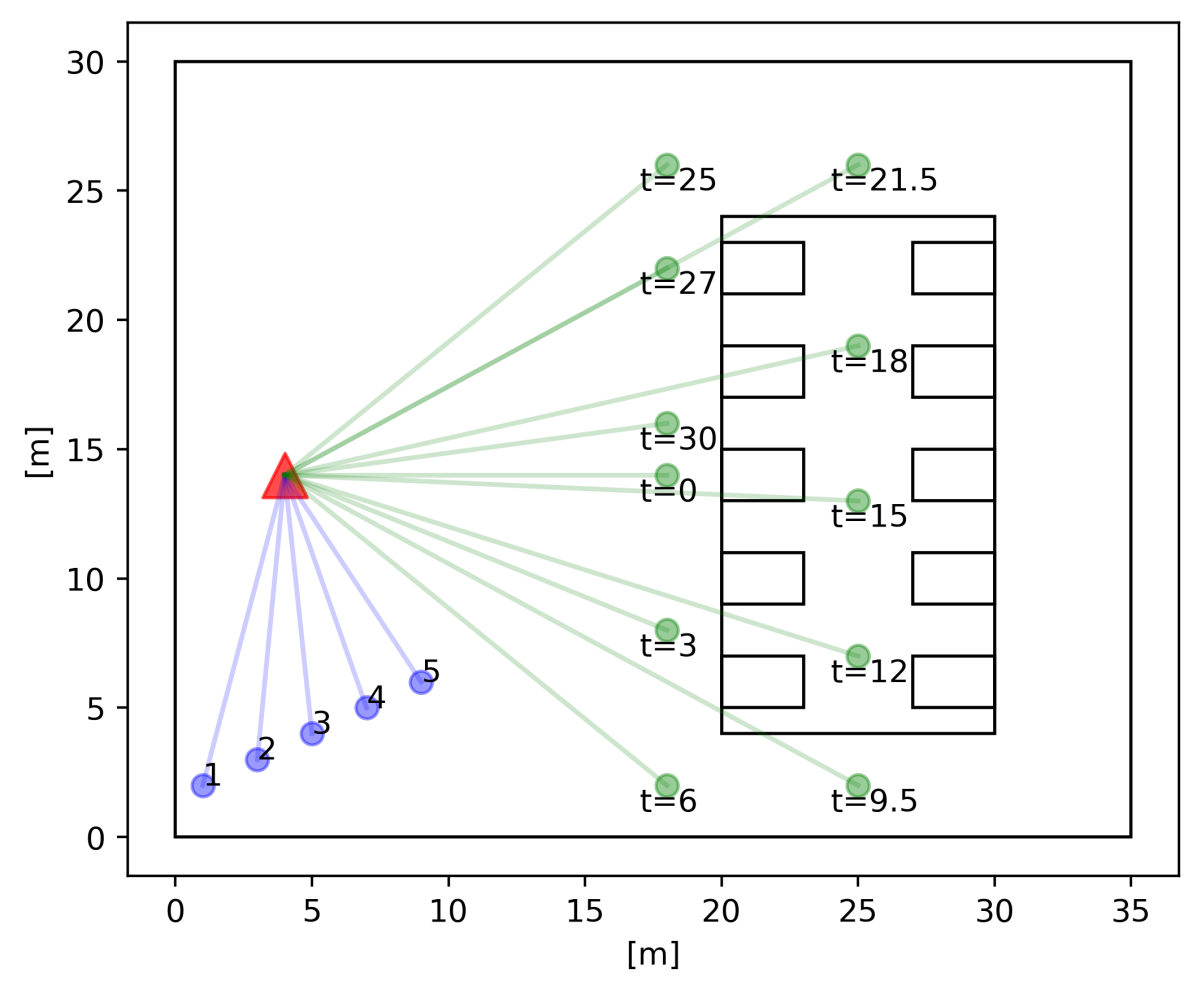
Simulation scenario. The red triangle represents the gNB position, the blue dots are the UEs that emulate the cell load, while the green dots represent the moving AGV. *t* denotes the time in seconds during the simulation, where the AGV is moving with a speed of 2 m/s.

The simulation scenario is shown in Figure 2. This scenario consists of a subsection of an indoor industry scenario. This subsection represents a stock storage area, where one automated guided vehicle (AGV) is moving to transport stock, as it is one of the main functions of AGVs. First, there is a remote host that is connected via a 100 Gb/s point-to-point connection to the Evolved Packet Core (EPC). This connection does not present propagation delay. Attending to the radio access network (RAN), a single 5G picocell with a height of 10 m is used, which will be shared by several users. There are six UEs connected to the picocell, where five of them generate background traffic to emulate a loaded cell environment. To do this, the remote host sends User Data Protocol (UDP) packet flows of 750 Mb/s for each one, with the aim of congesting the cell. These UEs have a fixed position and close to the next generation NodeB (gNB), therefore, they will have LOS conditions. On the other hand, the remaining UE has no fixed position, it moves and tries to emulate a remotely controlled AGV, which needs low latency. In this case, the remote host sends UDP packets with a periodicity of 100 ms and fixed size, as indicated in the simulation. Simulations have been made with packet sizes of 64 and 1000 bytes. These packet sizes represent two use cases when the remote host sends orders for controlling the AGV. The first one, with packet size of 64 bytes, corresponds to a packet that contains a unique order to the AGV. The second one, corresponds to a packet with several multiplexed orders. These packet sizes have been selected to evaluate the delay distribution obtained by each numerology.

To evaluate the NLOS condition, the AGV enters a room constructed of concrete with windows, with a height of 6 m. Inside, there are several concrete blocks with a height of 3 m that represent pallets and stock storage. These blocks are represented in Figure 2 as rectangles. In this figure, the movement of the AGV is also detailed, where *t* denotes the time in seconds during the simulation.

### 3.3. Simulation Parameters

We compare NR numerologies, from 0 to 4, and analyze the UDP end-to-end delay at PDCP layer, for the AGV remote-control use case, under full load condition with different packet sizes and channel condition. This delay is measured from the instant the gNB sends the PDCP protocol data unit (PDU) to the RLC layer until this PDU is received in the UE at PDCP level. Once received, the delay is calculated using the timestamps attached in the packet header. The main configuration parameters of the simulations are shown in Table 2.

We repeat the same simulations using 40 different random seeds for each packet size and channel condition, in order to obtain statistically significant results. Then, we aggregate all the results with different seeds in a boxplot.

## 4. Evaluation Results

This section shows the results obtained for each numerology and packet size over several iterations. Although the standard defines the use of different numerologies for each FR, as mentioned in Section 2, this study will analyze all numerologies in FR2 and if its application within this range for URLLC services is feasible. The motivation for choosing FR2 for study is that millimeter-wave bands can potentially boost capacity, reduce latency and provide a higher bandwidth. The analysis will focus on outliers, to better understand the behavior of the tail in the distribution of latency. Since URLLC communications are critical, it is important to understand and be able to reduce these outliers.

### 4.1. Results with Packet Size of 64 Bytes

**Figure 3:**
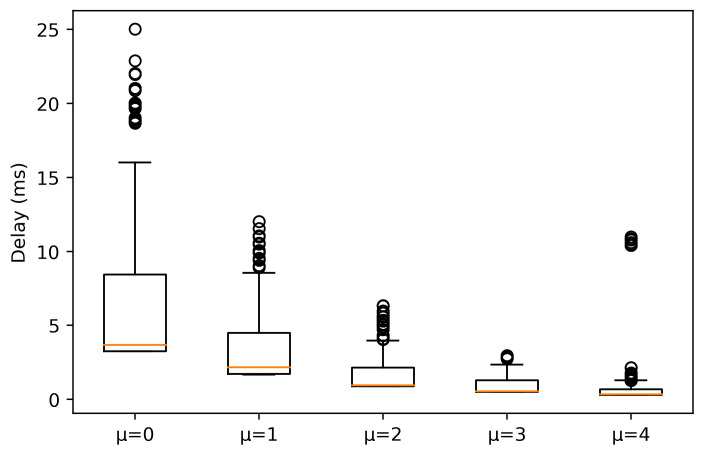
Experienced delay for packets with a size of 64 bytes in LOS conditions.

**Figure 4:**
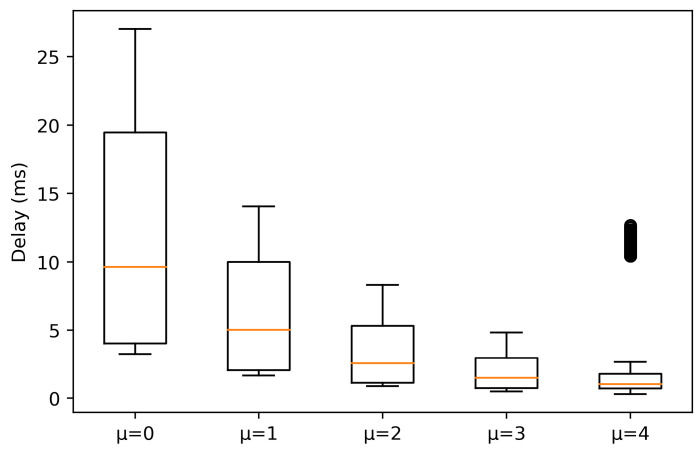
Experienced delay for packets with a size of 64 bytes in NLOS conditions.

Figure 3 and Figure 4 show the delay experienced by the packets when the AGV is under LOS and NLOS conditions, respectively. Under LOS conditions, it is observed that the higher μ is, the lower the delay. This was expected, since as μ increases, the slot duration is short, so the scheduling operation is faster. In this case, the median values for μ = {0, 1, 2} are 3.456, 1.778 and 0.956 ms, respectively. On the other hand, for μ = {3, 4} the median values are 0.536 and 0.336 ms, respectively. However, outliers exist for μ = 4, reaching values above 10 ms.

**Figure 5:**
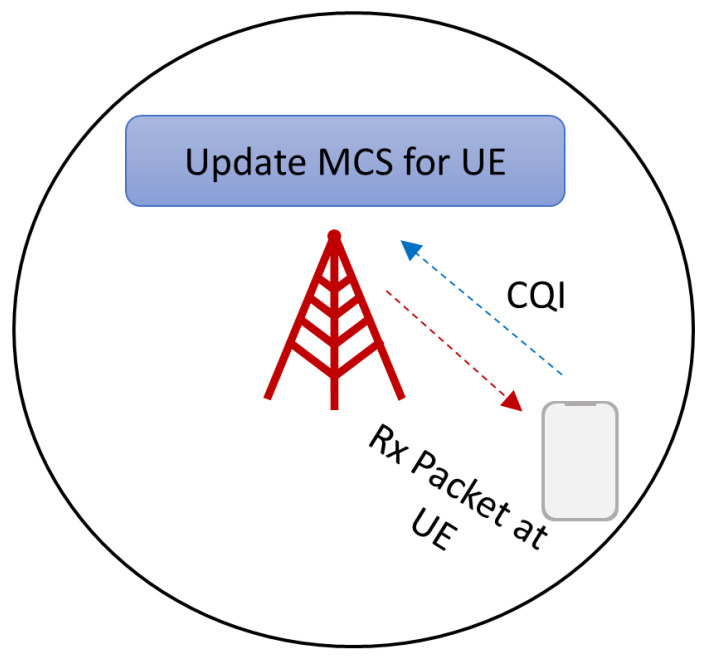
Modulation Coding Scheme (MCS) selection for downlink (DL) transmission.

Outliers are originated by two main factors. The first one is that as the AGV moves away from the gNB, the received signal-to-interference noise ratio (SINR) decreases, causing a more robust modulation selection. The selection of the modulation coding scheme (MCS) is done based on channel quality indicator (CQI) as shown in Figure 5. Upon a packet reception at the UE side, the UE measures the average SINR received for each packet chunk and then, based on this measure, the UE selects a CQI value, which is a scalar value from 0 to 15 that indicates how good or bad was the reception. Afterwards, the UE sends the CQI value to the gNB. When the gNB receives the CQI, it updates the MCS to be used in the next allocation for this UE according to CQI value. A robust MCS produces an increase in the delay, as OFDM symbols carry less bits, so it will be necessary more symbols to be allocated. The second one is related to the cell load level, where upon the arrival of a packet at MAC layer it may be the case of not having enough resources to allocate all the data in the current slot, having to wait for the next slot to allocate the rest of the data. The reason this occurs is because all traffic is treated fairly, i.e., there are no preferences for one traffic over another in the scheduler decision.

**Figure 6:**
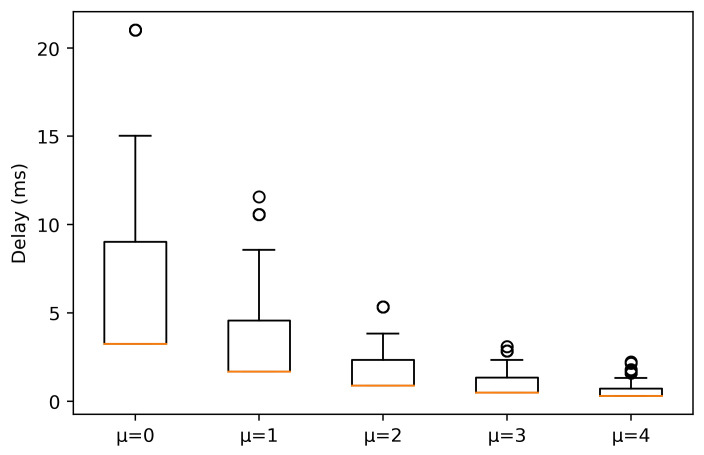
Experienced delay for packets with a size of 64 bytes in LOS conditions without background traffic.

**Figure 7:**
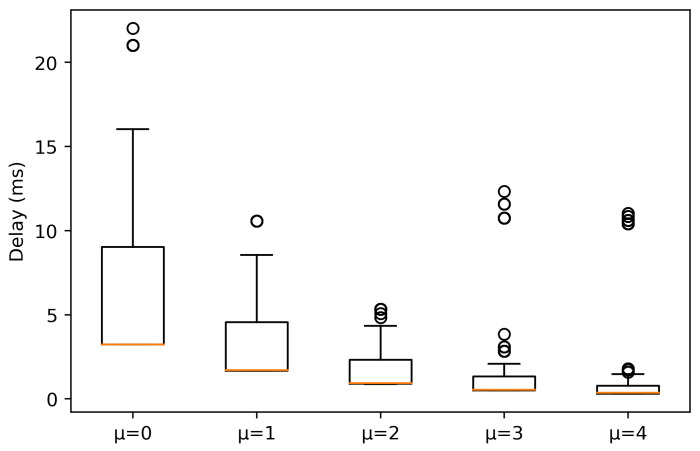
Experienced delay for packets with a size of 1000 bytes in LOS conditions without background traffic.

To check the effect of traffic background, we performed a simulation without that traffic that will help to understand why outliers occur with traffic background. Figure 6 and Figure 7 show the delay distribution when there is no traffic background in LOS conditions for packet sizes of 64 and 1000 bytes. As it can be seen, a higher μ provides a lower latency in both cases. Also, outliers are clearly reduced, since they are originated only by the changes of the modulation, due to the interference and SINR decrease. On the one hand, with 64 bytes, a packet arrival at MAC layer will always have enough resources, since these are not shared with other users. Therefore, the impact of the scheduler in allocating the resources between the different traffics is higher than the fact of transmitting with a more robust modulation. On the other hand, with 1000 bytes, the modulation scheme selected will have a higher impact on the delay for μ = {3, 4}, since the symbol duration is short and, if a robust modulation is selected, the OFDM symbol will carry less bits, so more symbols will be needed to allocate all the data. We do not repeat simulations without traffic background for the rest of the cases, since the trend is similar and this is an ideal case, cause in a real environment the network will not be empty.

Going back to Figure 3, μ = 3 maintains the delay more stable, as 25% and 75% percentile are very close to the median. There is a remarkable asymmetry in the values for each numerology, i.e., the 25% percentile is very close to the median, contrary to the 75% percentile. As numerology decreases, this distance goes further. This indicates that the values above the median present higher variation and that the time waiting for the next slot allocation is higher as μ decreases, due to having a slot with a longer duration. Thus, the extra delay introduced by waiting for next slots allocation will affect more for lower numerologies.

In the case of NLOS conditions, in Figure 4, it is shown that the delay experienced in the numerologies suffers more alterations, increasing, due to propagation losses and signal reflections. This is reflected in the median values obtained for each numerology, higher than in LOS condition. Again, a higher μ implies a lower delay, although there are outliers for μ = 4 between 10 and 15 ms. Moreover, it is observed that for μ = 3 the values do not increase significantly, and they remain stable and low, as 25% and 75% percentile are close to the median. However, for the rest of numerologies, there are major changes in the delay values. As numerology decreases, there is much more variation in the delay and, in contrast to LOS, the 25% and 75% percentile tends to be symmetric about the median.

### 4.2. Results with Packet Size of 1000 Bytes

**Figure 8:**
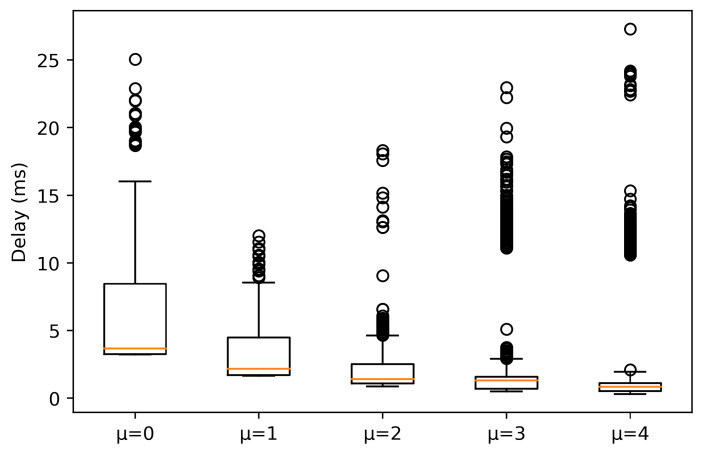
Experienced delay for packets with a size of 1000 bytes in LOS conditions.

**Figure 9:**
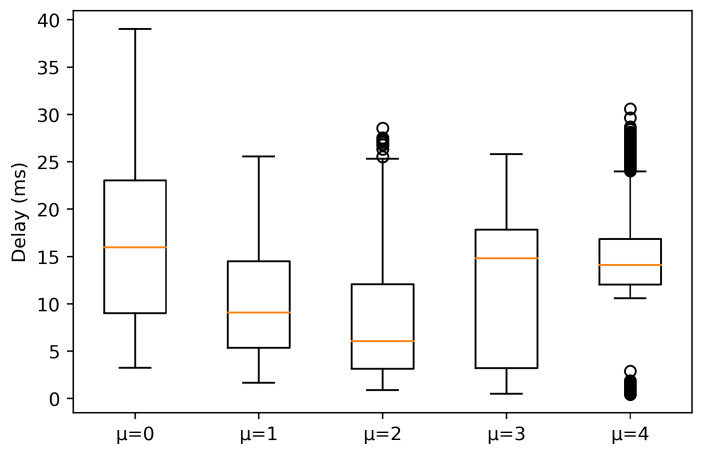
Experienced delay for packets with a size of 1000 bytes in NLOS conditions.

In the case of 1000 bytes packets size, the results obtained are shown in Figure 8 and Figure 9. As it can be observed, in LOS conditions, the trend of the values is similar to the case of packets with a size of 64 bytes for μ = {0, 1}, obtaining a median value of 3.669 and 2.169 ms, respectively. The main difference is that for μ = {2, 3, 4}, as numerology increases, although the median value decreases, there are outliers that differ more from the 75% percentile. These outlier values have a higher impact on the delay as μ increases. With shorter slots, the packet information cannot be scheduled in a single slot, more slots are needed to allocate all the information. Also, the UEs with traffic flows of 750 Mb/s accentuate this delay, as they also need resources that cannot be allocated in one slot. Thus, for next slots allocation, this will occur again, increasing the system delay.

On the other hand, under NLOS conditions, significant differences are observed. A remarkable difference is a high increase in the median for all numerologies, being more accentuated for μ = {3, 4}. Please note that for μ = 4, the 25% and 75% percentile tends to be symmetric about the median. However, outliers exist, reaching values above 25 ms and below 5 ms. On the other hand, for μ = 3, the 25% and 75% percentile tends to be asymmetric about the median. This clearly indicates that the data below the median present higher variation. Under this condition, μ = 2 obtains a median value of 6.041 ms, although there are outliers between 25–30 ms. Also, μ = 1 presents a similar behavior as μ = 2, but with a higher median value (9.062 ms). Finally, for μ = 0, it can be observed that the 25% and 75% percentile tends to be symmetric about the median and the median value is the highest. This indicates that this numerology is not suitable for AGV control, due to the delay distribution, where the 25% percentile is around 10 ms, which is not desirable for URLLC.

### 4.3. Result Discussion

On the one hand, it has been proven that with a higher μ in LOS conditions, the packet delay is lower, so shorter slots produce an apparent improvement, especially, with a small packet size. However, for μ = 4 the delay seems to be more unstable than μ = 3, due to a very short OFDM symbol duration. As adaptive modulation is used (the AGV distances itself from the gNB even though it has LOS), it may be the case that a more robust modulation is selected. The following occurs: if a more robust modulation is used, less information fits in a symbol and if that symbol has a very short duration in time (due to a very high numerology), more symbols are needed to be able to schedule all the packet information. Thus, the delay increases. The same happens when the packet size is increased for μ = {2, 3, 4}, as more symbols are required to transmit the packet data.

On the other hand, under NLOS conditions, a higher μ is not always suitable. This conclusion should be taken into account when using it for URLLC. Detection of LOS/NLOS, could help to select the μ according to radio conditions. With a small packet size, an improvement in the delay at high μ values is achieved, with more stable values for μ = 3 than μ = 4. When the packet size increases, better results are obtained with μ = 2 than μ = {3, 4}. In this case it is observed that an intermediate value for μ is more efficient than a high value under a loaded cell condition. This reflects that there is a balance between throughput and delay purposes, cause the slot duration is reduced but not too much, so the delay will be reduced without affecting too much in terms of throughput, as one OFDM symbol is large enough to be able to transmit the data and the queue size will be reduced in the scheduler.

## 5. Conclusions

In this paper, a comparison of the different numerologies proposed in the 3GPP NR standard in an industrial scenario is presented. It has been proven that not always a higher numerology provides a lower delay; it will depend on packet size and channel conditions. When a low packet size is selected, the premise that with higher μ the delay is lower is fulfilled under LOS and NLOS conditions, except for μ = 4 which presents outliers above 10 ms and a far distance from the median. This indicates that a very high slot time reduction cannot be suitable under high cell load conditions.

On the other hand, when the packet size increases, higher values of μ increase the delay. This is because when the slot is reduced, the information that can be scheduled in a single slot is also reduced, and the rest of the data must be allocated in other slots. This is important in industrial scenarios, where NLOS conditions are very common. Consequently, it will be necessary to complement the numerology selection with other mechanisms to service URLLC applications and reduce outliers, such as preemptive scheduling and resource reservation.

As a continuation of this work, multi-link connectivity will be investigated, which will provide higher reliability and, in certain cases, lower latency together with a good numerology selection.

## Figures and Tables

**Table 1:** Numerologies defined in 5G.

μ	SCS (kHz)	Slots per Subframe	Slot Duration (ms)	Symbol Duration (s)	Symbols per Slot
0	15	1	1	71.42	14
1	30	2	0.5	35.71	14
2	60	4	0.25	17.85	14
3	120	8	0.125	8.92	14
4	240	16	0.0625	4.46	14

**Table 2:** Main configuration parameters.

Parameter	Value
Channel and propagation loss model	3GPP 38.900
Channel condition	LOS and NLOS
System Bandwidth	200 MHz
Center frequency	28 GHz
Scenario	Indoor
Transmission Direction	Downlink
Modulation	Adaptive
Scheduler	Round-Robin
UE height	1.5 m
gNB height	10 m

## Data Availability

Not applicable.

## Abbreviations

The following abbreviations are used in this manuscript:

4G	Fourth generation
5G	Fifth generation
3GPP	Third Generation Partnership Project
AGV	Automated Guided Vehicle
BWP	Bandwidth Part
CQI	Channel Quality Indicator
DFT-s-OFDM	Direct Fourier Transform spread OFDM
eMBB	Enhanced Mobile BroadBand
EPC	Evolved Packet Core
ERP	Enterprise Resource Planning
FR	Frequency Range
gNB	Next generation NodeB
IoT	Internet of Things
LOS	Line-of-Sight
LTE	Long-Term Evolution
MAC	Media access control
MCS	Modulation Coding Scheme
MES	Manufacturing Execution System
mMTC	Massive Machine-Type Communications
mmWave	Millimeter-wave
NLOS	Non-Line-of-Sight
NR	New Radio
OFDM	Orthogonal Frequency Division Multiplexing
PAPR	Peak-to-Average-Power-Ratio
PDCP	Packet Data Convergence Protocol
PDU	Protocol Data Unit
PLC	Programmable Logic Controller
RAN	Radio Access Network
RLC	Radio Link Control
SCS	SubCarrier Spacing
SINR	Signal-to-interference noise ratio
TDD	Time Division Duplex
TDMA	Time Division Multiple Access
UDP	User Datagram Protocol
UE	User Equipment
URLLC	Ultra-Reliable and Low Latency Communications
WAN	Wide Area Network
